# Nonbacterial Thrombotic Endocarditis of the Aortic Valve Secondary to Urothelial Carcinoma With Pancreatic Metastases

**DOI:** 10.7759/cureus.94472

**Published:** 2025-10-13

**Authors:** Alex P Rodriguez, Sonia I Vicenty-Rivera

**Affiliations:** 1 Cardiology, Bruce W. Carter Department of Veterans Affairs Medical Center, Miami, USA; 2 Research and Development, Veterans Affairs Caribbean Healthcare System, San Juan, PRI

**Keywords:** aortic valve, cardiac magnetic resonance, cardiac metastasis, nonbacterial endocarditis, transthoracic echocardiography, urothelial carcinoma

## Abstract

A 72-year-old male with a medical history significant for ischemic cardiomyopathy, prostate cancer, and peripheral vascular disease presented with jaundice, progressive frailty, and anemia. Transthoracic echocardiography identified a new aortic valve vegetation. Comprehensive infectious evaluation yielded negative results, and cardiac magnetic resonance (CMR) imaging demonstrated a non-enhancing lesion involving the aortic valve.

Given the negative infectious evaluation and absence of systemic signs of infection, a diagnosis of nonbacterial thrombotic endocarditis (NBTE) was favored. Concurrent imaging revealed pancreatic involvement by metastatic urothelial carcinoma. Anticoagulation therapy was initiated but subsequently discontinued due to the development of symptomatic anemia and thrombocytopenia.

This case underscores the diagnostic utility of CMR in characterizing valvular lesions and emphasizes the importance of malignancy screening in patients with suspected NBTE. A multidisciplinary approach enabled the formulation of a patient-centered management strategy within the context of advanced malignancy.

## Introduction

The diagnosis of endocarditis is primarily based on the modified Duke criteria. However, distinguishing between infectious endocarditis (IE) and non-infectious etiologies remains challenging. Multimodality imaging plays a pivotal role in the diagnostic evaluation of most cases. Nonbacterial thrombotic endocarditis (NBTE), a rare condition, is typically associated with hypercoagulable states, autoimmune conditions, and malignancies [1].

The aim of this article is to provide a comprehensive review of current diagnostic approaches to endocarditis, with a specific focus on differentiating between infectious and non-infectious etiologies, and to highlight the clinical and imaging features of NBTE.

## Case presentation

A 72-year-old man was admitted for evaluation of progressive frailty, painless jaundice, and profound symptomatic anemia. His medical history was significant for chronic ischemic cardiomyopathy, coronary artery disease, diabetes mellitus, chronic kidney disease, peripheral artery disease with a proximal femoral artery stent, prostate carcinoma with associated bone metastasis, and high-grade papillary urothelial carcinoma treated with nephroureterectomy. Even though the patient had prostate cancer with cone metastases post radiation with undetectable prostate-specific antigen levels. Initial laboratory studies demonstrated marked elevations in aminotransferases (aspartate aminotransferase (AST) 406 U/L and alanine aminotransferase (ALT) 330 U/L) and bilirubin (total 12.9 mg/dL, direct 9.4 mg/dL).

Exams and imaging

**Figure 1:**
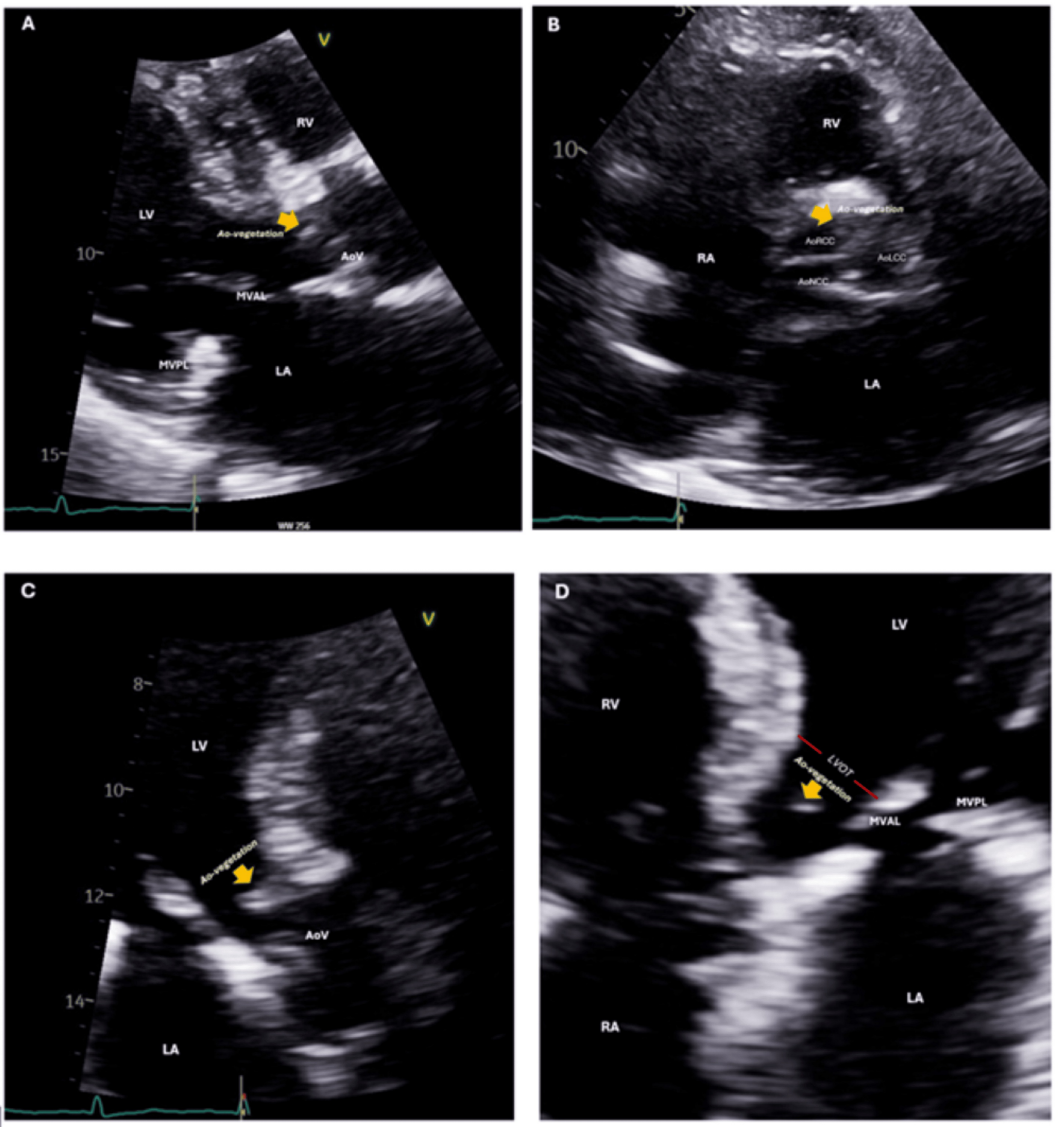
Two-dimensional transthoracic echocardiography (Panel A: parasternal long-axis view, Panel B: parasternal short-axis view, Panel C: apical three-chamber view, and Panel D: apical five-chamber view) demonstrates a trileaflet aortic valve with leaflet thickening but preserved systolic excursion. Notably, a sclerotic, prolapsing structure is visualized between the right and left coronary cusps, raising suspicion for valvular vegetation (yellow arrow). LA: left atrium RA: right atrium LV: left ventricle MVAL: mitral valve anterior leaflet MVPL: mitral valve posterior leaflet LVOT: left ventricular outflow tract

Transthoracic echocardiography on admission questioned the possibility of a new, prominent vegetation on the aortic valve prolapsing into the left ventricular outflow tract (LVOT) during diastole seen on different views (Figure 1). Infectious Disease, Cardiology, and Cardiothoracic Surgery (CTS) teams were consulted. An extensive evaluation for infective endocarditis was undertaken, ultimately excluding an infectious etiology. Additional laboratory findings included normocytic anemia and thrombocytopenia.

Multiple blood and urine cultures remained negative for over two weeks. The workup for IE was extensive and included testing for Mycobacterium tuberculosis, fungal pathogens, Coxiella burnetii, Bartonella species, and HACEK organisms (Haemophilus species, Aggregatibacter, Cardiobacterium, Eikenella, and Kingella). Throughout the hospitalization, the patient remained hemodynamically and clinically stable, afebrile, and without leukocytosis, with no compelling evidence supporting an infectious etiology. Both Cardiothoracic Surgery and Cardiology consultants agreed that the findings were more consistent with a non-infectious process.

**Figure 2:**
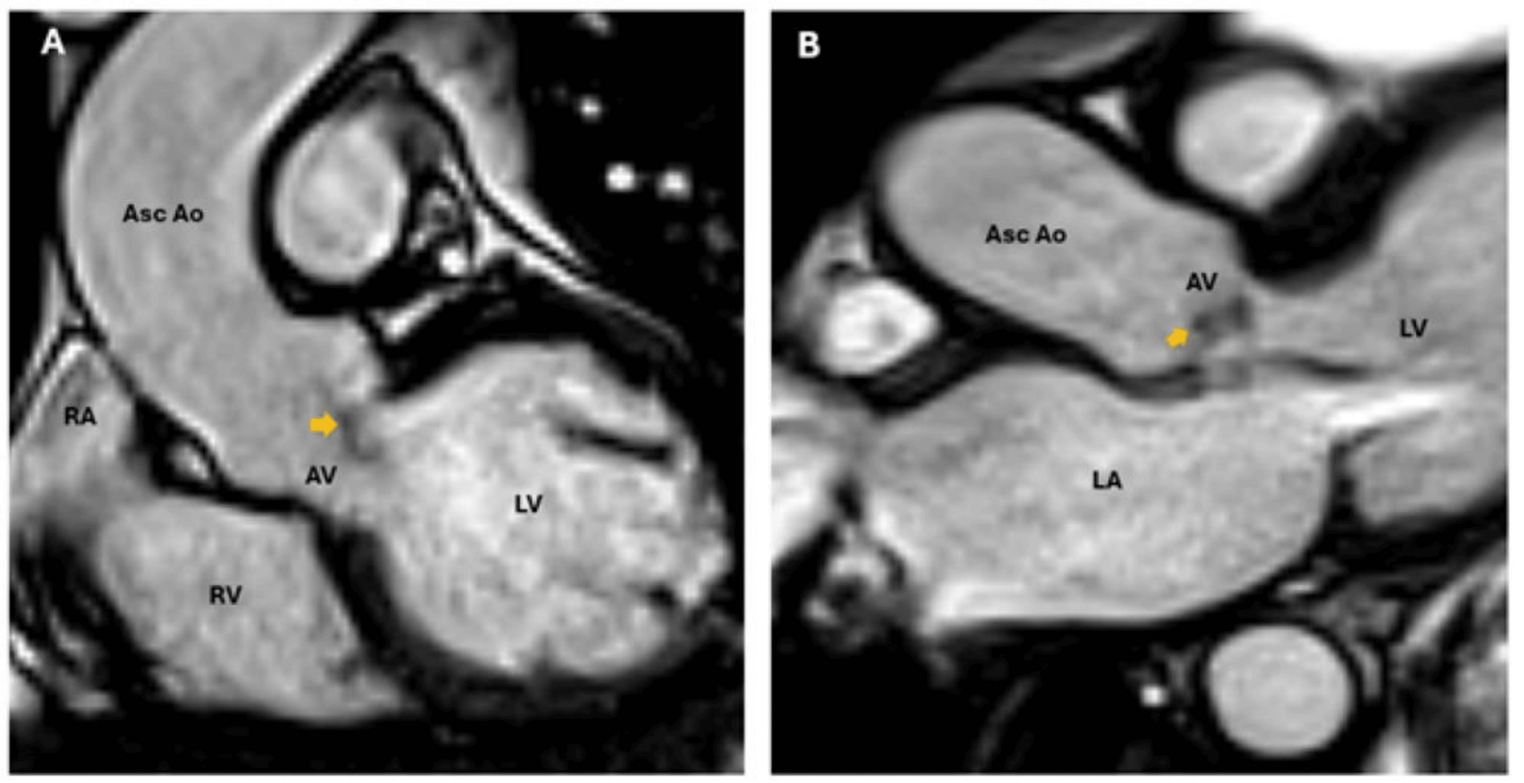
Cardiac MRI three-chamber views (Panels A and B) demonstrate an aortic valve with leaflet thickening and a vegetation adherent to one of the aortic cusps (yellow arrow). LA: left atrium RA: right atrium LV: left ventricle RV: right ventricle AV: aortic valve Asc Ao: ascending aorta

**Figure 3:**
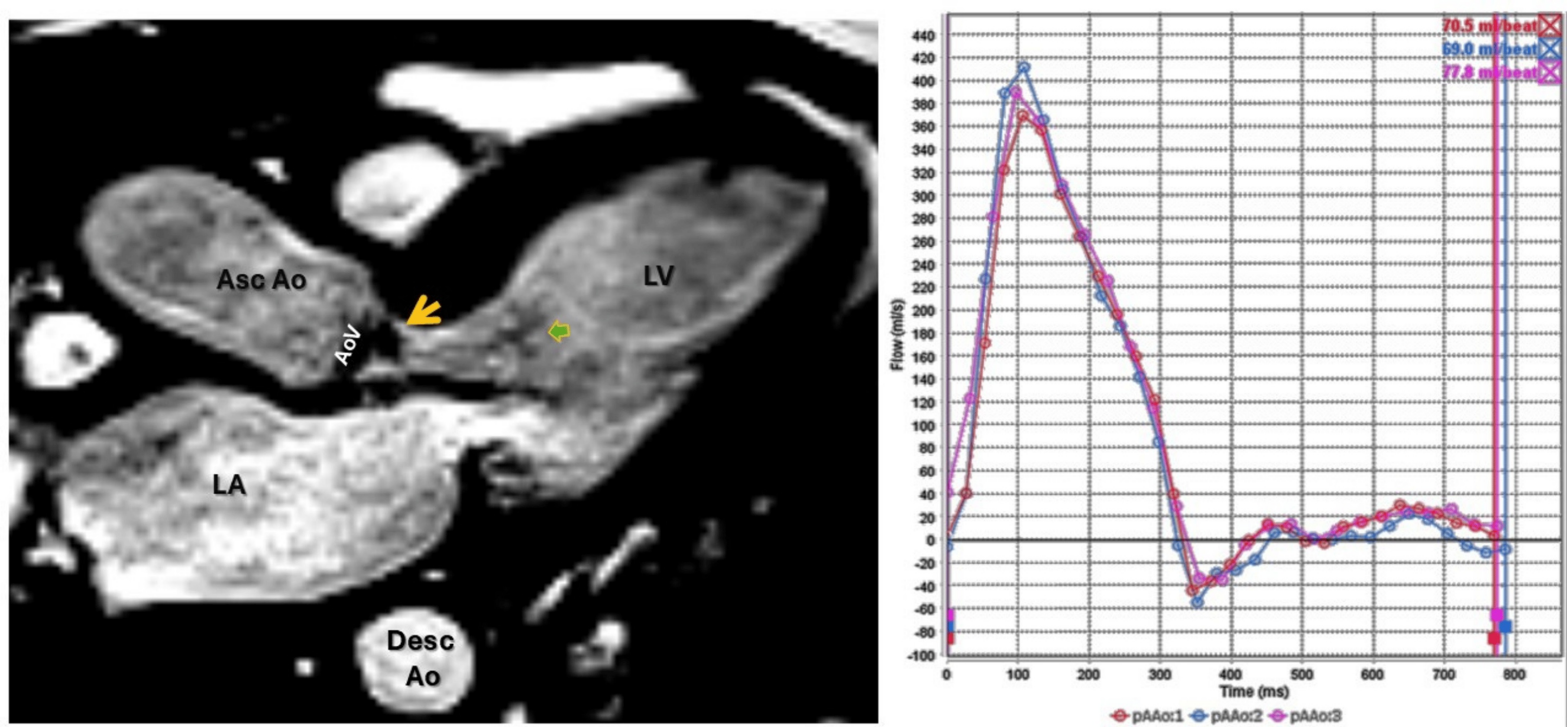
Cardiac magnetic resonance (CMR) three-chamber view (left panel) demonstrates thickened aortic valve cusps with associated aortic regurgitant flow originating from the right coronary cusp and extending into the left ventricular outflow tract (LVOT) (yellow arrow), accompanied by evidence of aortic regurgitation (green arrow). The right panel displays flow sequences, which reveal no significant aortic regurgitation. LV: left ventricle LA: left atrium AoV: aortic valve Asc Ao: ascending aorta Desc Ao: descending aorta

**Figure 4:**
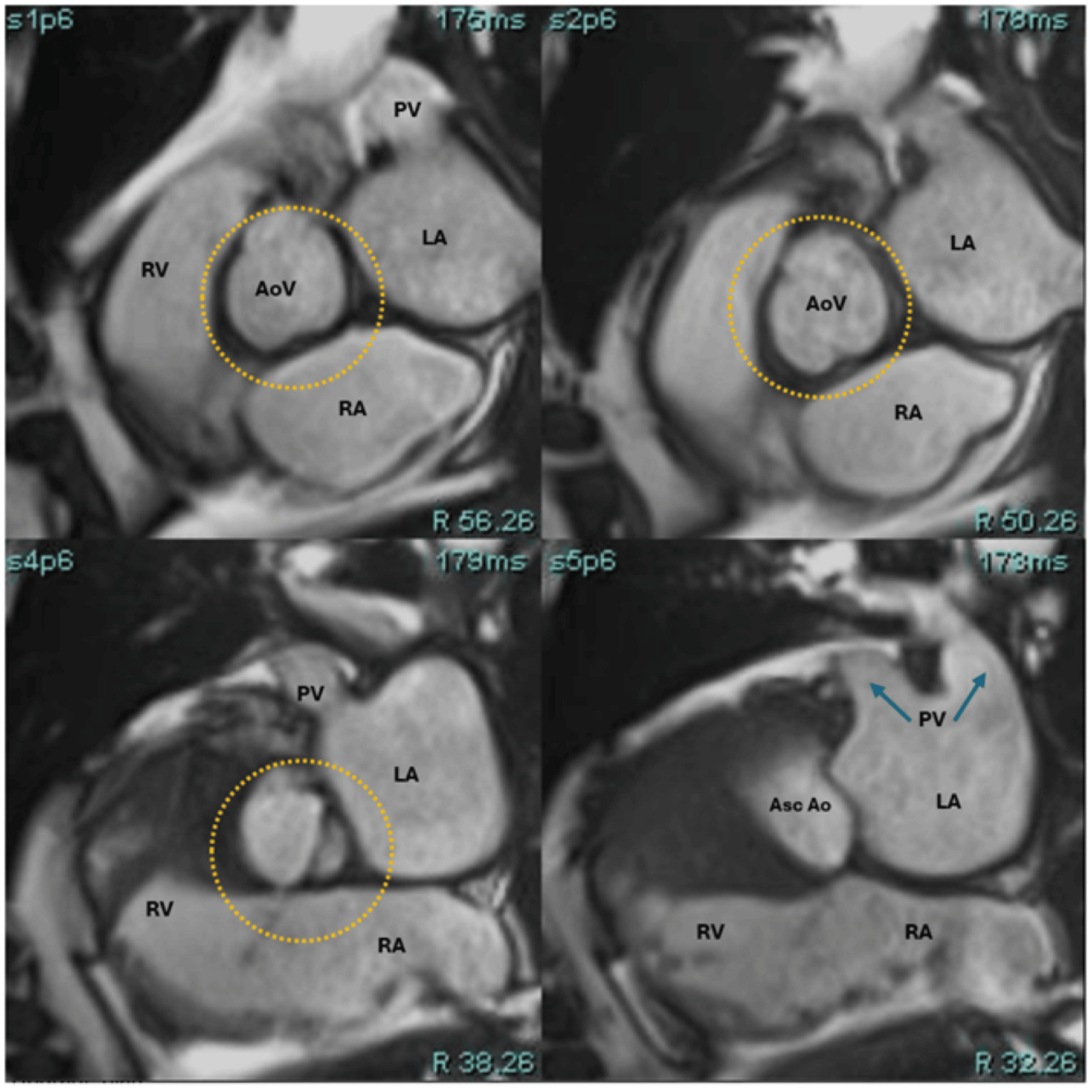
Cardiac magnetic resonance (CMR) assessment of the aortic valve and annulus on orthogonal valve-planned cine images without evidence of perivalvular abscess, valvular perforation, or rupture (yellow dashed circle). PV: pulmonary veins AoV: aortic valve LA: left atrium RA: right atrium LV: left ventricle RV: right ventricle

The patient was evaluated by anesthesiology, and due to acute liver injury and obstructive jaundice, a transesophageal echocardiogram (TEE) was deferred. Instead, a cardiac magnetic resonance (CMR) study was subsequently obtained, confirming the presence of a large, non-enhancing vegetation on the right coronary cusp of the aortic valve, prolapsing into the LVOT during diastole (Figures 2). Flow sequences demonstrated no significant aortic regurgitation (Figure 3); in addition, there was no evidence of perivalvular abscess, valvular perforation, or rupture (Figures 4). The CMR also revealed severe biventricular dilatation and severe left ventricular systolic dysfunction. In the first pass, resting perfusion did not reveal any mass enhancement. T1 and T2 map excluded any infiltrative process or myocardial edema.

Further evaluation with abdominal and pelvic imaging identified an obstructive lesion at the head of the pancreas. Magnetic resonance cholangiopancreatography (MRCP) raised concern for underlying malignancy, and subsequent endoscopic retrograde cholangiopancreatography (ERCP) confirmed the diagnosis of high-grade urothelial carcinoma with pancreatic metastasis.

Treatment plan

A biliary stent was placed, resulting in marked improvement of the previously elevated liver enzymes. Cytology biliary stricture brushing performed during biliary stent placement disclosed metastatic high-grade urothelial carcinoma.

A multidisciplinary and comprehensive evaluation, including heart team, oncology, urology, and palliative care, recommended the initiation of anticoagulation. Subsequently, it was discontinued due to the development of significant, symptomatic anemia - requiring multiple transfusions - and worsening thrombocytopenia. The patient and family actively participated in detailed discussions regarding the risks and benefits of further diagnostic evaluation and respective therapeutic interventions, ultimately guiding a patient-centered approach to care.

Patient outcomes

The patient and his family elected hospice care and continued to follow up with all consultants on an outpatient basis until his demise.

## Discussion

NBTE refers to sterile, non-infective vegetations composed primarily of platelet-fibrin aggregates on previously undamaged cardiac valves, most commonly affecting the mitral and aortic valves [1,2]. It is usually associated with advanced malignancies (especially adenocarcinomas), systemic lupus erythematosus (SLE), antiphospholipid syndrome, and hypercoagulable states [2]. Differentiating NBTE from infective endocarditis remains a significant clinical challenge, particularly in patients with complex comorbidities and atypical presentations. NBTE is a diagnosis of exclusion and should be considered in patients with valvular vegetations and negative infectious workups, especially in the context of malignancy. NBTE in cancer is a paraneoplastic hypercoagulable state characterized by sterile fibrin-platelet vegetations attached to heart surfaces, such as valves, with the potential to embolize to the brain. It is most often linked to advanced adenocarcinomas (i.e., pancreas, lung, gastrointestinal tract, and ovary) [2]. It arises from a malignancy-driven hypercoagulable state, leading to endothelial injury and cytokine-mediated platelet activation, which produces friable, sterile fibrin-platelet vegetations that can readily embolize distally to other organs. Clinical presentation in NBTE can be variable; however, patients frequently present with systemic embolic events such as strokes, myocardial infarctions, renal and/or splenic infarcts, and pulmonary emboli, among others [3,4].

The objective of this article is to provide a comprehensive and critical appraisal of current diagnostic methodologies for endocarditis, with a specific focus on the differentiation between infectious and non-infectious causes. Furthermore, this review aims to delineate the clinical and imaging characteristics of NBTE, and to underscore the utility of advanced multimodality imaging in enhancing diagnostic precision and informing evidence-based clinical decision-making [5,6]. 

This case highlights a rare instance of NBTE secondary to pancreatic metastasis from primary urothelial carcinoma. CMR was instrumental in excluding intracardiac malignancy or metastases, comprehensive evaluation of valvular abnormalities, and ruling out structural complications, ultimately guiding individualized clinical decision-making [6]. Additionally, CMR contributed to broader oncologic assessment and cardiovascular risk stratification, supporting a comprehensive, tailored approach to care. Lately, CMR has emerged as a useful adjunct for NBTE diagnosis, showing the capability in detecting and characterizing valvular masses and associated inflammation. Although transesophageal echocardiography remains the diagnostic standard imaging modality due to higher sensitivity and specificity for smaller vegetations [7-9], CMR may often provide ancillary information. First, CMR offers higher tissue characterization than echocardiography. Secondly, in patients suspected of malignancy, CMR is more adept at evaluating primary cardiac malignancies, metastases, and pericardial involvement. The latter is crucial in patients with suspected Libman-Sacks endocarditis [7,10,11].

CMR protocols for the evaluation of marantic endocarditis should be designed to maximize lesion detection and characterization, as well as to assess valvular anatomy, subvalvular and supravalvular complications, and myocardial and systemic involvement. A comprehensive CMR protocol should include high-resolution cine imaging, T1 and T2 parametric mapping, flow sequences, and late gadolinium enhancement (LGE) imaging [11,12]. Cine imaging provides a dynamic assessment of valvular morphology and function, enabling the detection of valvular masses and their effects on leaflet motion, such as restriction, perforation, and the mechanisms underlying regurgitation or obstruction. Parametric mapping facilitates the identification of myocardial edema and inflammation, which is particularly important in the evaluation of autoimmune disorders that may present with myocarditis, pericarditis, or both. LGE imaging, preferably using phase-sensitive inversion recovery (PSIR) sequences, enables detection of hyperenhancement. Typically, avascular structures, such as vegetations in bacterial endocarditis, do not demonstrate gadolinium enhancement. However, in some cases of acute NBTE, associated edema and ongoing fibrosis may result in delayed enhancement. This may aid in differentiating sterile thrombotic vegetations from other types of lesions [11,12].

Other pertinent valvular lesions, such as papillary fibroelastomas, myxomas, and fibromas, also require careful evaluation for exclusion. CMR can be particularly helpful and informative in this context [7,12,13]. In such cases, a thorough assessment of adjacent anatomy is essential. CMR enables detailed evaluation of lesion morphology, dimensions, location, extent, homogeneity, adjacent tissue infiltration, and tissue characteristics. Tissue characterization is pivotal for identifying or excluding features such as necrosis, hemorrhage, calcification, vascularity, and fatty infiltration. First-pass gadolinium perfusion imaging may also be used to assess mass vascularity or its absence. In our case, we demonstrated that the mass was avascular by the absence of contrast enhancement during first-pass imaging.

Despite its strengths, CMR has several limitations in evaluating valvular vegetations that warrant consideration. First, compared to echocardiography, CMR has lower temporal resolution [11]. Second, it is susceptible to various artifacts, including signal loss in areas of high-velocity, non-laminar flow [12], which can complicate the assessment of vegetations and adjacent anatomy. Third, the relatively long duration of CMR protocols may be challenging for patients with claustrophobia, potentially leading to suboptimal compliance and technically limited studies. Finally, many patients may have relative or absolute contraindications to undergoing MRI, in which case the examination may need to be deferred or canceled altogether.

This case also illustrates the critical importance of a comprehensive and methodical diagnostic evaluation. Initially, imaging and clinical presentation suggested a primary pancreatic malignancy as the source of the patient’s obstructive jaundice and systemic illness [13]. However, a detailed workup, including advanced imaging and tissue sampling, ultimately revealed the true underlying diagnosis: high-grade urothelial carcinoma with pancreatic metastasis. This distinction had significant implications for prognosis, management decisions, and discussions of the goals of care. The complexity of this diagnostic pathway underlines the need for persistent investigation beyond initial impressions, particularly in patients with atypical presentations and overlapping clinical features.

NBTE remains a rare but clinically significant entity that demands a high index of suspicion, particularly in patients with malignancy and culture-negative endocarditis. Our case illustrates the diagnostic complexity of NBTE, especially when classical diagnostic tools, such as TEE, pose an impermissibly high risk. It also highlights the critical role of multimodality imaging, particularly cardiac magnetic resonance CMR, in detecting valvular abnormalities, excluding alternative diagnoses, assessing structural complications, and contributing to comprehensive cardiovascular and oncologic risk stratification. CMR’s advanced tissue characterization capabilities proved instrumental in guiding clinical decision-making and supporting a patient-centered, multidisciplinary approach to care [12].

**Figure 5:**
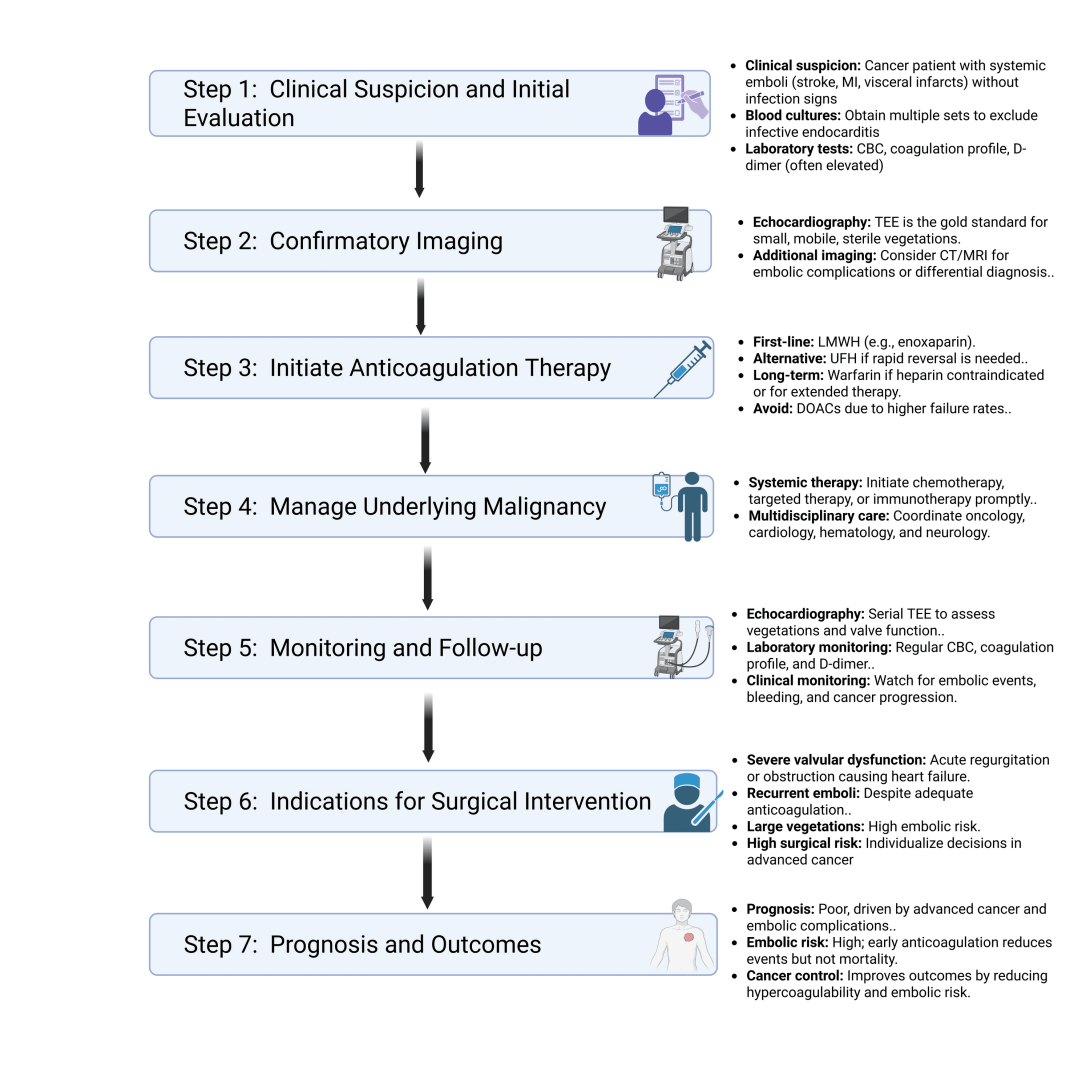
Management of Cancer Related Nonbacterial Endocarditis MI: myocardial infarction TEE: transesophageal echocardiography LMWH: low molecular weight heparin UFH: unfractionated heparin DOAC: direct oral anticoagulant

Diagnosis and management of NBTE require a comprehensive infectious workup, exclusion of alternative causes, interdisciplinary collaboration, and careful risk-benefit assessment when considering anticoagulation in patients at increased risk for embolic events (Figure 5). Cancer-related NBTE is associated with a poor prognosis, primarily due to advanced cancer and embolic complications. Management involves heparin-based anticoagulation and cancer therapies to reduce hypercoagulability, with valve replacement surgery reserved for refractory cases with severe valvular dysfunction or recurrent embolic events despite anticoagulation [14]. This case underscores the importance of early recognition and individualized management in improving outcomes for this vulnerable population.

## Conclusions

In conclusion, NBTE remains a challenging diagnosis that requires a high index of suspicion, especially in patients with malignancy and culture-negative endocarditis. Cardiac magnetic resonance imaging is an invaluable tool in differentiating NBTE from other cardiac and metastatic lesions, assessing structural complications, and informing clinical management. A multidisciplinary, patient-centered approach, rooted in thorough diagnostic evaluation and early, targeted intervention, is essential for improving outcomes in this complex patient population. The objective of this article is to deliver a rigorous and scholarly evaluation of contemporary diagnostic approaches to endocarditis, with particular emphasis on distinguishing infectious from non-infectious etiologies. Specifically, this review aims to systematically characterize the clinical presentation and imaging features of NBTE and to critically evaluate the role of advanced multimodality imaging in enhancing diagnostic accuracy and supporting evidence-based management strategies.
